# A20 Curtails Primary but Augments Secondary CD8^+^ T Cell Responses in Intracellular Bacterial Infection

**DOI:** 10.1038/srep39796

**Published:** 2016-12-22

**Authors:** Sissy Just, Gopala Nishanth, Jörn H. Buchbinder, Xu Wang, Michael Naumann, Inna Lavrik, Dirk Schlüter

**Affiliations:** 1 Institute of Medical Microbiology and Hospital Hygiene, Otto-von-Guericke University Magdeburg, 39120 Magdeburg, Germany; 2 Organ-specific Immune Regulation, Helmholtz-Center for Infection Research, 38124 Braunschweig, Germany; 3 Department of Translational Inflammation Research, Otto-von-Guericke University Magdeburg, 39106 Magdeburg, Germany; 4 Institute of Experimental Internal Medicine, Otto-von-Guericke University Magdeburg, 39120 Magdeburg, Germany

## Abstract

The ubiquitin-modifying enzyme A20, an important negative feedback regulator of NF-κB, impairs the expansion of tumor-specific CD8^+^ T cells but augments the proliferation of autoimmune CD4^+^ T cells. To study the T cell-specific function of A20 in bacterial infection, we infected T cell-specific A20 knockout (CD4-Cre A20^fl/fl^) and control mice with *Listeria monocytogenes*. A20-deficient pathogen-specific CD8^+^ T cells expanded stronger resulting in improved pathogen control at day 7 p.i. Imaging flow cytometry revealed that A20-deficient *Listeria*-specific CD8^+^ T cells underwent increased apoptosis and necroptosis resulting in reduced numbers of memory CD8^+^ T cells. In contrast, the primary CD4^+^ T cell response was A20-independent. Upon secondary infection, the increase and function of pathogen-specific CD8^+^ T cells, as well as pathogen control were significantly impaired in CD4-Cre A20^fl/fl^ mice. *In vitro*, apoptosis and necroptosis of *Listeria*-specific A20-deficient CD8^+^ T cells were strongly induced as demonstrated by increased caspase-3/7 activity, RIPK1/RIPK3 complex formation and more morphologically apoptotic and necroptotic CD8^+^ T cells. *In vitro*, A20 limited CD95L and TNF-induced caspase3/7 activation. In conclusion, T cell-specific A20 limited the expansion but reduced apoptosis and necroptosis of *Listeria*-specific CD8^+^ T cells, resulting in an impaired pathogen control in primary but improved clearance in secondary infection.

CD8^+^ T cells are key players for the elimination of intracellular bacterial and viral pathogens. Upon stimulation with antigen, naïve pathogen-specific CD8^+^ T cells undergo rapid clonal expansion and effector CD8^+^ T cells (T_eff_) move to the site of infection, where they produce protective effector molecules and kill infected cells^1^. Following the peak of the primary CD8^+^ T cell response, typically around day 7, a contraction phase is induced, leading to massive cell death of T_eff_ 2. Only 5–10% of the original pool of pathogen-specific CD8^+^ T cells survive and form a pool of long-living memory CD8^+^ T cells (T_mem_)^3^. Upon rechallenge with the same pathogen, CD8^+^ T_mem_ cells rapidly expand and provide improved protection. The processes of (i) naïve CD8^+^ T cell activation and expansion, (ii) effector CD8^+^ T cell death during the contraction phase, and (iii) development and survival of memory CD8^+^ T cells are controlled by the NF-κB pathway, which is activated upon stimulation of the T cell receptor (TCR) by cognate antigen^4^,^5^,^6^,^7^. In addition, CD4^+^ T cells help during the acute phase of listeriosis by supporting the development of memory CD8^+^ T cells^8^,^9^.

A tight regulation of NF-κB activation is crucial for controlling the magnitude of the CD8^+^ T cell response and the development of different pathogen-specific CD8^+^ T cell subsets^10^. In intracellular bacterial infection, reduced activation of NF-κB results in impaired expansion of primary pathogen-specific CD8^+^ T cells^11^. However, prolonged stimulation of NF-κB also diminishes primary CD8^+^ T cell expansion by promoting their caspase-8 expression and apoptosis^5^. Interestingly, increased apoptosis of CD8^+^ T cells is mediated by CD95/CD95L interaction, which also regulates the development and maintenance of effector memory (T_EM_) versus central memory (T_CM_) CD8^+^ T cells^5^,^12^.

The ubiquitin modifying enzyme A20 plays a pivotal role as a negative feedback regulator of the canonical NF-κB signaling pathway upon activation of various cytokine and pattern recognition receptors^13^,^14^,^15^. A20 limits NF-κB activation by removing K63-linked polyubiquitin chains from signaling molecules including receptor-interacting serine/threonine-protein kinase 1 (RIPK1), RIPK2, TNF receptor-associated factor 6 (TRAF6) and IKK-γ (NEMO)^16^,^17^,^18^,^19^. In addition, A20 builds K48-polyubiquitin chains on RIPK1 inducing proteasomal degradation and indirectly inhibits signaling pathways by the suppression of NF-κB-induced gene expression^16^,^20^. A20 has also been reported to control the cell death program, in particular, of RIPK1/RIPK3-dependent necroptosis, by blocking ubiquitination of the RIPK1/3 complex^21^.

A20-deficient mice die prematurely of cachexia and tissue inflammation due to a hyperactivation of NF-κB^22^. In addition, selective deletion of A20 in dendritic cells^23^,^24^,^25^, myeloid cells^26^, B cells^27^,^28^,^29^, and astrocytes^20^ respectively, results in or exacerbates autoimmunity. In humans, polymorphisms of the A20-encoding *Tnfaip3* gene predisposes to numerous diseases including autoimmune disorders^30^. In contrast, T cell-specific deletion of A20 protects from CD4^+^ T cell-mediated autoimmunity, which may be partially explained by increased cell death of activated A20-deficient CD4^+^ T cells due to caspase-independent and RIPK3-dependent necroptosis^21^. On the contrary, Giordano *et al*. have shown that deletion of A20 in T cells induced a more efficient anti-tumor CD8^+^ T cell response without evidence for an increased cell death of activated A20-deficient T cells^31^.

Here we investigated the role of A20 in the regulation of the CD8^+^ T cell response during intracellular bacterial infection. Upon infection with *Listeria monocytogenes* (Lm), T cell-specific A20 limited the magnitude of the primary effector CD8^+^ T cell response resulting in an impaired pathogen control. Notably, A20 reduced apoptosis and necroptosis of Lm-specific CD8^+^ T cells during primary T cell response, promoted survival of T_mem_ and improved protection against secondary infection.

## Results

### T cell numbers and activation in naïve mice

CD4-Cre A20^fl/fl^ mice were born in normal Mendelian ratio and survived without any clinical signs of disease for at least one year (data not shown). In good agreement with Onizawa *et al*.^21^, splenic CD4^+^ and CD8^+^ T cells of CD4-Cre A20^fl/fl^ mice were normal in numbers but slightly more activated, as indicated by increased CD69 expression, in comparison to A20^fl/fl^ mice (Supplementary Fig. S1a–c). The small reduction of CD4^+^ T cell numbers may be caused by a reduction of invariant NK T cells, since A20 is required for the development of this subset of CD4^+^ T cells^32^.

### Improved control of acute systemic listeriosis in CD4-Cre A20^fl/fl^ mice

**Figure 1:**
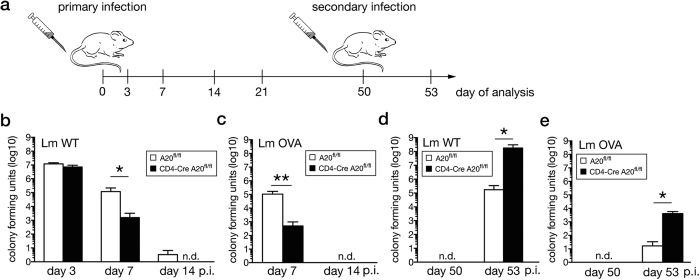
Improved control of *Listeria monocytogenes* during primary infection but impaired clearance upon rechallenge in CD4-Cre A20^fl/fl^ mice. (**a**) Experimental design: CD4-Cre A20^fl/fl^ and A20^fl/fl^ control mice were infected with Lm and spleens were analyzed at the indicated time points. Reinfection was performed 50 days after the primary infection. (**b**) CFU in spleen was determined at day 3, 7 and 14 after infection with Lm WT. (**c**) CFU in spleen after Lm OVA infection was determined at day 7 and 14 p.i. (**d**) CFU in spleen of Lm WT infected mice at day 50 and 3 days after reinfection at day 53. (**e**) CFU in spleen of Lm OVA infected mice at day 50 and 3 days after reinfection at day 53. Data are compiled of 3 independent experiments with 3-5 animals per group and experiment. Error bars indicate + SEM. Non-parametric Mann Whitney test, with *p < 0.05, **p < 0.01.

Since CD8^+^ T cells play a pivotal role in the clearance of Lm, we studied the effect of T cell-specific A20 on (i) pathogen control and (ii) the expansion and effector function of pathogen-specific CD8^+^ T cells in primary and secondary listeriosis according to the experimental outline illustrated in Fig. 1a.

Upon primary infection with wildtype (Lm WT) and ovalbumin-expressing Lm (Lm OVA), pathogen control was significantly improved in CD4-Cre A20^fl/fl^ mice in spleen (Fig. 1b,c) and liver (data not shown) at day 7 p.i. Up to day 50 p.i., Lm WT and Lm OVA were eliminated from spleens of both mouse strains. In sharp contrast to primary infection, reinfection on day 50 p.i. resulted in an impaired control of Lm WT and Lm OVA in CD4-Cre A20^fl/fl^ mice (Fig. 1d,e).

**Figure 2:**
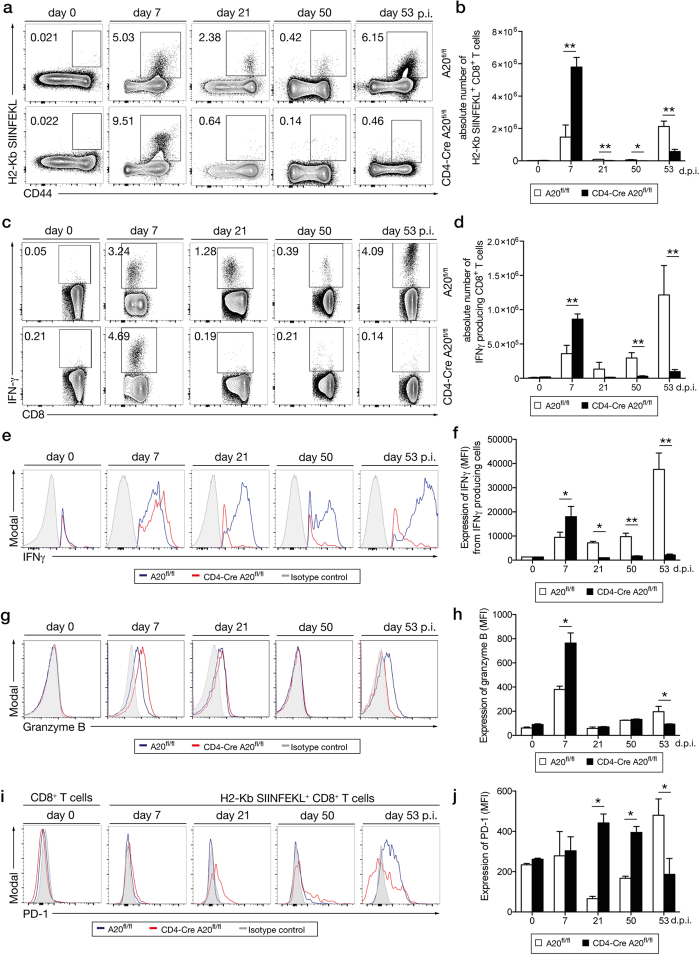
Improved primary but impaired secondary CD8^+^ T cell response in CD4-Cre A20^fl/fl^ mice. CD4-Cre A20^fl/fl^ and A20^fl/fl^ control mice were infected with a non-lethal dose of Lm OVA and CD8^+^ T cell response in spleen was analyzed at the indicated time points. (**a**) Representative dot plots and (**b**) absolute number of H2-Kb SIINFEKL pentamer^+^ CD8^+^ T cells after primary infection (day 0, 7 and 21 p.i.) and after reinfection (day 50 and 53 p.i.) with Lm OVA. (**c**) Representative dot plots and (**d**) absolute number of IFN-γ producing CD8^+^ T cells p.i. with Lm OVA and *ex vivo* restimulation with SIINFEKL peptide for 4 h in the presence of Brefeldin A. (**e**) IFN-γ-producing CD8^+^ T cells were gated and representative histograms of IFN-γ is displayed for the indicated time points after Lm OVA infection. (**f**) IFN-γ MFI from IFN-γ-producing CD8^+^ T cells. (**g**) Representative histograms and (**h**) granzyme B MFI of CD8^+^ T cells after Lm OVA infection and *ex vivo* restimulation with SIINFEKL peptide. (**i**) Representative histograms and (**j**) MFI of PD-1 expression on bulk CD8^+^ T cells (0 d.p.i.) or Lm OVA-specific CD8^+^ T cells (7, 21, 50 and 53 d.p.i.). A representative of 3 independent experiments is shown with 3 mice per group. Error bars indicate + SEM. Student’s *t*-test, *p < 0.05.

In accordance with the kinetics of pathogen control, the relative and absolute numbers of Lm OVA-specific CD8^+^ T cells were significantly increased in CD4-Cre A20^fl/fl^ mice at day 7 after infection with Lm OVA, i.e. the peak of the primary CD8^+^ T cell response (Fig. 2a,b). In contrast, the numbers of Lm OVA-specific IFN-γ-producing CD4^+^ T cells were identical in both mouse strains (Supplementary Fig. S3a) In parallel to pathogen clearance, Lm OVA-specific CD8^+^ T cells declined in both mouse strains gradually up to day 50 p.i. (Fig. 2a,b). However, this decline was stronger in CD4-Cre A20^fl/fl^ mice and, upon secondary infection, the increase of Lm OVA-specific CD8^+^ T cells was significantly impaired as compared to A20^fl/fl^ control mice. Upon reinfection of A20^fl/fl^ control mice, the absolute number of pathogen-specific CD8^+^ T cells was increased as compared to the primary response. The number of pathogen-specific CD8^+^ T cells in CD4-Cre A20^fl/fl^ mice, however, was reduced compared to the peak of the primary response (Fig. 2b).

Consistently, relative and absolute numbers of IFN-γ-producing A20-deficient Lm OVA-specific CD8^+^ T cells were increased at day 7 p.i. (Fig. 2c,d). The numbers of protective IFN-γ-producing Lm OVA-specific CD8^+^ T cells declined over time in both mouse strains but IFN-γ-positive Lm OVA-specific CD8^+^ T cells were significantly reduced in CD4-Cre A20^fl/fl^ at day 50 p.i. Importantly, the mean fluorescence intensity (MFI) of IFN-γ-producing Lm OVA-specific CD8^+^ T cells was significantly increased in CD4-Cre A20^fl/fl^ mice at day 7 p.i. but rapidly declined thereafter resulting in a reduced IFN-γ production as compared to control mice at days 21 and 50 p.i. (Fig. 2e,f). Upon secondary infection, the relative and absolute numbers of IFN-γ-producing Lm OVA-specific CD8^+^ T cells strongly increased in A20^fl/fl^ mice, whereas absolute numbers only slightly expanded in CD4-Cre A20^fl/fl^ mice (Fig. 2c,d). Importantly, the IFN-γ MFI enhanced strongly in reinfected A20^fl/fl^ mice but weakly increased in CD4-Cre A20^fl/fl^ mice (Fig. 2e,f).

The kinetics of granzyme B-producing CD8^+^ T cells complemented the characteristic of the IFN-γ kinetics with increased numbers and MFI of granzyme B^+^ cells in CD4-Cre A20^fl/fl^ mice in primary infection at day 7 p.i. but reduced numbers and MFI in reinfected mice at day 53 p.i. (Fig. 2g,h, Supplementary Fig. S2).

The reduced IFN-γ and granzyme B MFI of A20-deficient CD8^+^ T cells indicated a functional impairment in addition to reduced expansion. Therefore, we determined the expression of PD-1, which is upregulated on activated CD8^+^ T cells and can limit the function of pathogen-specific CD8^+^ T cells^33^. As illustrated in Fig. 2i,j, PD-1 was expressed on bulk CD8^+^ T cells in uninfected mice and also equally expressed on Lm OVA-specific CD8^+^ T cells of both strains at day 7 p.i. Thereafter, A20-competent Lm OVA-specific CD8^+^ T cells strongly downregulated PD-1 expression, whereas it further increased in A20-deficient CD8^+^ T cells. Upon reinfection, PD-1 MFI of A20-sufficient Lm OVA-specific CD8^+^ T cells strongly increased but declined in A20-deficient CD8^+^ T cells (Fig. 2i,j).

Next, we analyzed the Lm OVA-specific CD4^+^ T cell response, since CD4^+^ T cell help during acute infection by regulating CD8^+^ T cell responses in listeriosis^8^,^9^. In contrast to CD8^+^ T cells, numbers of Lm OVA-specific IFN- γ-producing CD4^+^ T cells were equally increased in both mouse strains (Supplementary Fig. S3a). Obar *et al*. reported that the expansion of Lm-specific CD8^+^ T cells in primary listeriosis may also depend on CD4^+^ T cell mediated upregulation of CD25 on CD8^+^ T cells early after infection (day 5 to 7 p.i.)^34^. Therefore, we determined the expression of CD25 on CD8^+^ T cells in acute listeriosis. As illustrated in Supplementary Fig. 3c, CD8^+^ T cells of both mouse strains equally up-regulated CD25 expression up to day 7 p.i.

In conclusion, Lm-specific CD4^+^ T cell responses were not significantly regulated by A20 indicating that the A20-dependent reduction of the primary but augmentation of the secondary Lm-specific CD8^+^ T cell response were CD4^+^ T cell independent.

### A20 inhibits CD8^+^ T cell activation *in vitro*

*In vitro* stimulation of purified CD8^+^ T cells with anti-CD3/CD28 resulted in enhanced expression of A20 in control A20^fl/fl^ cells (Supplementary Fig. S4a). The inhibitory role of A20 for T cell activation was further confirmed by an increased IκBα phosphorylation of A20-deficient CD8^+^ T cells as well as a slightly increased phosphorylation of p38 and ERK (Supplementary Fig. S4a). In accordance with our *in vivo* data, activation of A20-deficient T cells was augmented as illustrated by increased IFN-γ and TNF production (Supplementary Fig. S4b) and enhanced proliferation (Supplementary Fig. S4c,d).

### Rapid decline of A20-deficient pathogen-specific effector, effector memory and central memory CD8^+^ T cells

**Figure 3:**
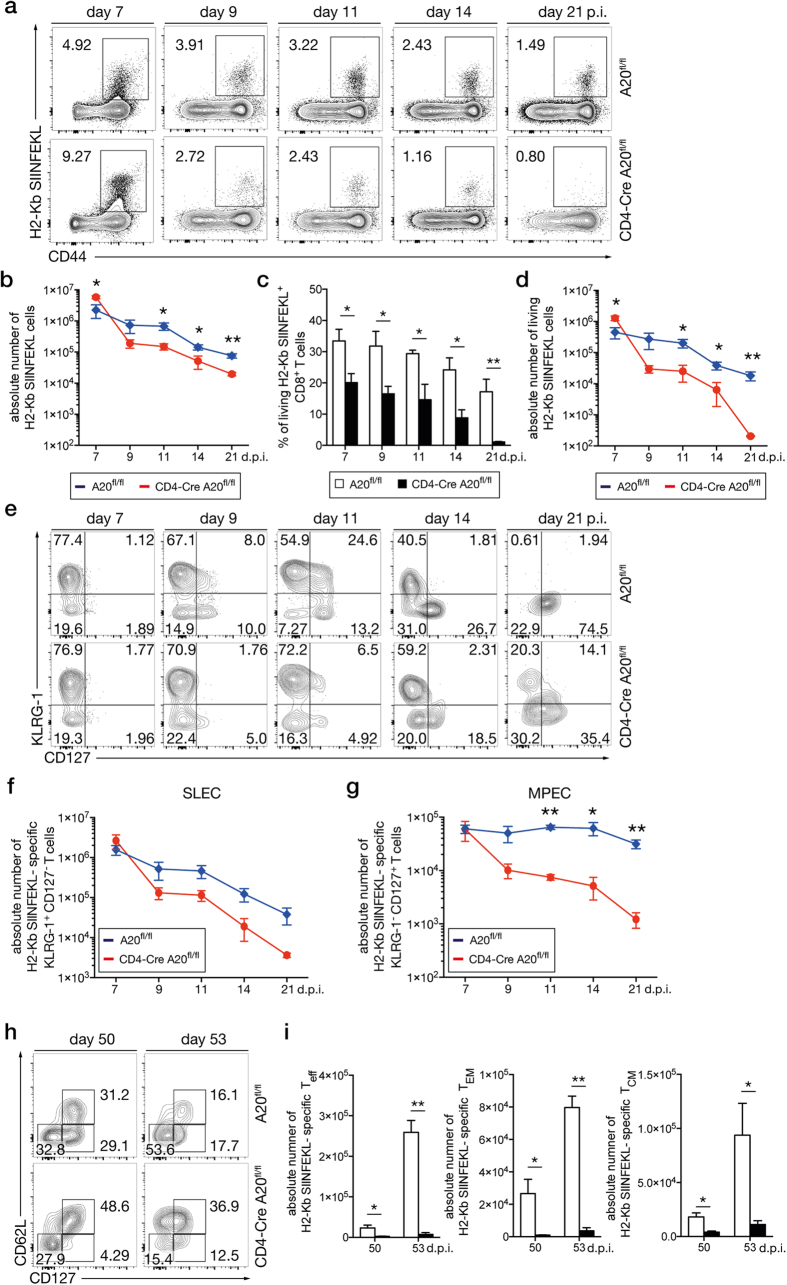
Reduced numbers of memory and memory-precursor CD8^+^ T cells in CD4-Cre A20^fl/fl^ mice. CD4-Cre A20^fl/fl^ and A20^fl/fl^ control mice were infected with a non-lethal dose of Lm OVA and spleens were analyzed for T_mem_ formation at the indicated time points. (**a**) Kinetic of Lm OVA-specific CD8^+^ T cells, stained with H2-Kb SIINFEKL pentamer and gated on singlets and CD3^+^ CD8^+^ cells. (**b**) Absolute numbers of total (living, apoptotic and necroptotic) Lm OVA-specific CD8^+^ T cells. (**c**) Relative numbers of living (annexin^−^/7-AAD^−^) Lm OVA-specific CD8^+^ T cells. (**d**) Absolute numbers of living (annexin^−^/7-AAD^−^) Lm OVA-specific CD8^+^ T cells. (**e**) Representative dot plots with frequencies of Lm OVA-specific CD8^+^ SLEC (KLRG-1^+^ CD127^−^) and MPEC (KLRG-1^−^ CD127^+^) were determined. (**f**) Absolute numbers of Lm OVA-specific CD8^+^ SLEC. (**g**) Absolute numbers of Lm OVA-specific CD8^+^ MPEC. (**h**) Representative dot plots with frequencies of T_eff_ (CD62L^low^ and CD127^low^), T_EM_ (CD62L^low^ and CD127^high^) and T_CM_ (CD62L^high^ and CD127^high^) were determined at day 50 and 53 p.i. (i) Absolute numbers of Lm OVA-specific CD8^+^ T_eff_, T_EM_ and T_CM_ cells. Data are compiled of 2 independent experiments with 3–5 animals per group and experiment. Error bars indicate + SEM. Student’s *t*-test, *p < 0.05; **p < 0.01.

Since CD4-Cre A20^fl/fl^ mice developed an improved early primary CD8^+^ T cell response but failed to elicit the secondary response, we defined the time point of the loss of pathogen-specific A20-deficient CD8^+^ T cells by performing a detailed kinetic of Lm OVA-specific CD8^+^ T cells. After the peak of the primary Lm OVA-specific CD8^+^ T cell response at day 7 p.i., the relative (Fig. 3a) and absolute (Fig. 3b) numbers of pathogen-specific CD8^+^ T cells decreased in both strains of mice but the decline was significantly stronger in CD4-Cre A20^fl/fl^ mice as compared to A20^fl/fl^ control mice. Already at day 9 p.i., numbers of pathogen-specific CD8^+^ T cells were lower in CD4-Cre A20^fl/fl^ mice. Thereafter, Lm OVA-specific CD8^+^ T cells declined with similar kinetic in both strains up to day 21 p.i., and absolute numbers of Lm OVA-specific CD8^+^ T cells remained consistently higher in A20^fl/fl^ mice.

Further, we determined the relative (Fig. 3c) and absolute (Fig. 3d) number of living Lm OVA-specific CD8^+^ T cells by 7-AAD/annexin V staining during primary infection. The relative number of living Lm OVA-specific CD8^+^ T cells was significantly reduced in CD4-Cre A20^fl/fl^ mice from day 7 to day 21 p.i. However, in CD4-Cre A20^fl/fl^ mice, the absolute number of living cells (7-AAD^−^ annexin V^−^) was significantly higher as compared to CD4-Cre A20^fl/fl^ mice at day 7 p.i. but, thereafter, declined stronger in CD4-Cre A20^fl/fl^ mice. At day 21 p.i., CD4-Cre A20^fl/fl^ mice harbored only a very small number of living Lm OVA-specific CD8^+^ T cells (Fig. 3d).

Antigen-experienced CD8^+^ T cells are a heterogeneous group consisting of KLRG-1^+^ CD127^−^ short-lived effector cells (SLEC) and KLRG-1^−^ CD127^+^ memory precursor effector cells (MPEC)^35^,^36^. Therefore, we further investigated the development and kinetics of these cells. The relative and absolute numbers of Lm OVA-specific SLEC and MPEC were similar in both mouse strains at day 7 p.i. (Fig. 3e–g). Thereafter, SLEC declined in both strains but the relative number of SLEC remained higher in CD4-Cre A20^fl/fl^ as compared to A20^fl/fl^ mice (20.3 vs. 0.61% at day 21 p.i.). In contrast to SLEC, relative numbers of MPEC increased in both mouse strains but the increase was substantially higher in A20^fl/fl^ mice (Fig. 3e). This resulted in a stable absolute number of MPEC in A20^fl/fl^ mice, whereas they declined over time in CD4-CreA20^fl/fl^ mice (Fig. 3g). Thus, at day 21 p.i., the Lm OVA-specific CD8^+^ T cell population of A20-sufficient animals contained mostly MPEC, while the A20-deficient Lm OVA-specific CD8^+^ T cell compartment consisted of a mixed population of SLEC and MPEC.

Next, we studied the relative and absolute numbers of T_eff_ (CD62L^−^ CD127^−^), T_EM_ (CD62L^−^ CD127^+^) and T_CM_ (CD62L^+^ CD127^+^) at day 50 after primary infection and three days after reinfection (d53). At day 50 p.i., relative numbers of T_EM_ were lower in CD4-Cre A20^fl/fl^ mice and absolute numbers of all three subpopulations were significantly reduced in CD4-Cre A20^fl/fl^ as compared to A20^fl/fl^ mice (Fig. 3h,i). Upon reinfection, T_eff_ increased strongly in A20^fl/fl^ mice with an additional lower proliferation of T_EM_ and T_CM_, whereas the increase of all CD8^+^ subpopulations was marginal in CD4-Cre A20^fl/fl^ mice (Fig. 3i).

Taken together, A20 strongly supported the survival of Lm OVA-specific CD8^+^ T cells and the development of MPEC, T_EM_ and T_CM_ CD8^+^ T cells resulting in a stronger expansion of T_eff_ upon reinfection.

### A20-deficiency enhances necroptosis and apoptosis of pathogen-specific CD8^+^ T cells

**Figure 4:**
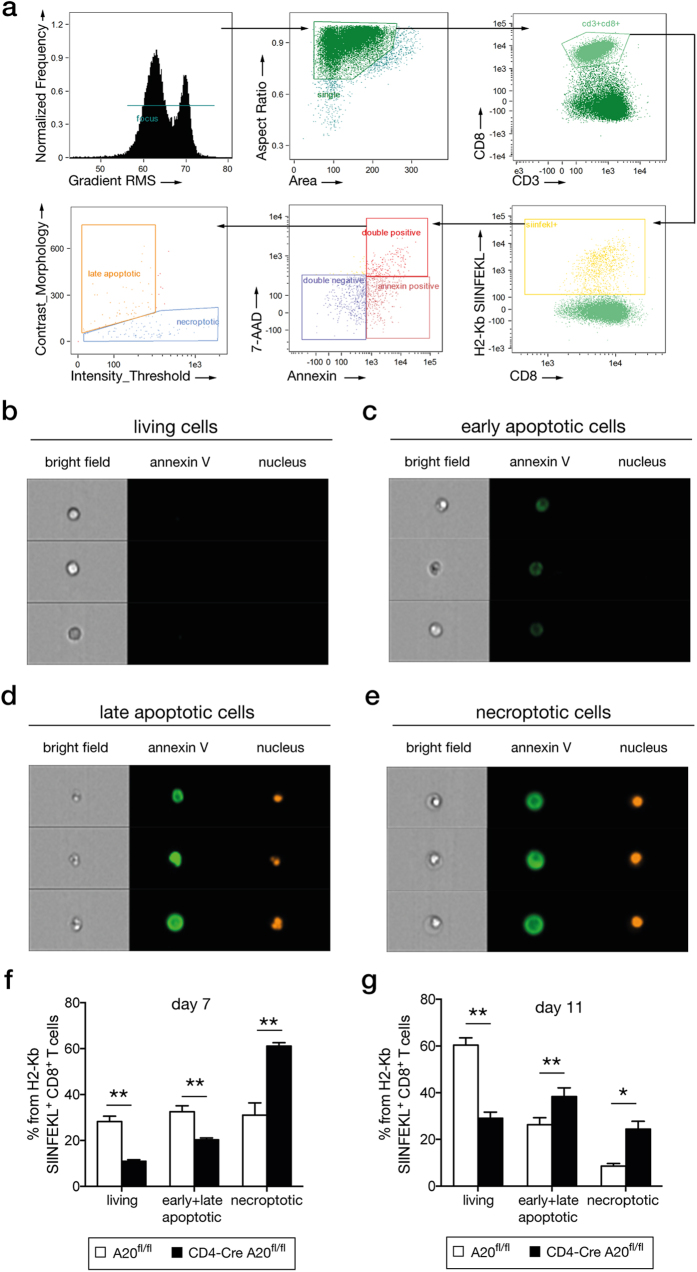
Enhanced necroptosis and apoptosis of A20-deficient CD8^+^ T cells. CD4-Cre A20^fl/fl^ and A20^fl/fl^ control mice were infected with a sub-lethal dose of Lm OVA. On day 7 and 11 p.i., Lm OVA-specific CD8^+^ T cells were analyzed with IFC for apoptotic and necroptotic morphology. (**a**) IFC gating strategy for Lm OVA-specific CD8^+^ T cells and determination of double negative, annexin V^+^ (early apoptotic), and annexin V/7-AAD double positive cells. Double positive cells were further distinguished into late apoptotic and necroptotic cells. (**b–e**) Examples of microscopical analysis of cell morphology with IFC. (**b**) Living cells: negative for annexin V and 7-AAD. (**c**) Early apoptotic cells: annexin V^+^/7-AAD^−^ with beginning of nucleus condensation and membrane blebbing. (**d**) Late apoptotic cells: annexin V^+^/7-AAD^+^ with condensed nuclei and membrane blebbing. (**e**) Necroptotic cells: annexin V^+^/7-AAD^+^ with swollen cell and nuclei. (**f–g**) Frequency of living, apoptotic (early and late stage) and necroptotic Lm OVA-specific CD8^+^ T cells in spleen at day 7 p.i. (**f**) and day 11 p.i. (**g**). A representative of 2 independent experiments is shown, with 3–4 mice per group. Error bars indicate + SEM. Student’s *t*-test, *p < 0.05; **p < 0.01.

The elimination of pathogen-specific CD8^+^ T cells was enhanced in CD4-Cre A20^fl/fl^ mice, therefore, we investigated how A20 influenced cell death pathways. Due to the most substantial loss of pathogen-specific CD8^+^ T cells on day 7 and 11 p.i., we focused on these time points for the cell death analysis p.i. (Fig. 3a,b). Two major cell death programs apoptosis and programmed necrosis, also termed necroptosis, control the fate of T cells^37^. There are major differences in the morphology of the apoptotic *vs.* necroptotic cells that allow to distinguish between these two types of cell death. In particular, the necroptotic cells are typically swollen, while apoptotic cells undergo shrinkage during the course of cell death^38^. The identification of Lm OVA-specific CD8^+^ T cells undergoing apoptosis or necroptosis was performed by imaging flow cytometry (IFC), allowing the simultaneous analysis of a single cell by flow cytometry and microscopy to differentiate apoptosis from necroptosis^38^. Figure 4a illustrates the gating strategy for Lm OVA-specific CD8^+^ T cells (CD3^+^ CD8^+^ OVA-pentamer^+^) and the analysis of living (7-AAD^−^ annexin V^−^), early apoptotic (7-AAD^−^ annexin V^+^) and late apoptotic/necroptotic cells (7-AAD^+^ annexin V^+^) cell. Nuclear and membrane morphology of these cells were used to differentiate between living (Fig. 4B), early apoptotic (Fig. 4c), late apoptotic (Fig. 4d) and necroptotic cells (Fig. 4e).

At day 7 p.i., the relative number of living 7-AAD^−^ annexin V^−^ pathogen-specific CD8^+^ T cells with normal cellular morphology was approximately 30% in control mice but only 10% in CD4-Cre A20^fl/fl^ mice (Fig. 4f). In control mice, equal percentages of apoptotic (7-AAD^+/−^ annexin V^+^ with condensed nuclei and normal cell membrane) and necroptotic (7-AAD^+^ annexin V^+^ with swollen cell size and nuclei) Lm OVA-specific CD8^+^ T cells were present at day 7 p.i. (Fig. 4f). In contrast, relative numbers of Lm OVA-specific apoptotic cells were significantly reduced whereas necroptotic pathogen-specific CD8^+^ T cells were significantly increased in CD4-Cre A20^fl/fl^ mice (Fig. 4f).

At day 11 p.i., relative numbers of Lm OVA-specific apoptotic and necroptotic cells were significantly increased in CD4-Cre A20^fl/fl^ mice, whereas an increase of living Lm OVA-specific CD8^+^ T cells was observed in A20^fl/fl^ mice (Fig. 4g).

Thus, the pool of pathogen-specific CD8^+^ T cells as shown in Figs 2a and 3b consists of living, apoptotic and necroptotic cells, and both cell death mechanisms contribute to the decline of pathogen-specific CD8^+^ T cells at the peak of the primary T cell response, which is controlled by A20.

### A20-deficiency enhances apoptosis and necroptosis of CD8^+^ T cells *in vitro*

Down modulation of the primary T cell response has been reported to occur via CD95L/CD95 system and involves the activation of caspase-8, which further activates executioner caspases, including caspase-3, leading to apoptosis of the cell^39^. In addition to CD95, stimulation with TNF contributes to activation of cell death pathways. Stimulation of death receptors can result in the activation of a caspase-independent cell death pathway by the interaction and activation of RIPK1 and RIPK3, inducing necroptosis. Previously, it has been reported that anti-CD3/CD28 stimulation of A20-deficient CD4^+^ T cells results in an increased caspase-independent RIPK1/RIPK3-dependent necroptosis^21^. Since we detected both apoptosis and necroptosis of pathogen-specific CD8^+^ T cells *in vivo*, we further analyzed the mechanisms of how A20 regulates the cell death pathways in naïve CD8^+^ T cells *in vitro*.

**Figure 5:**
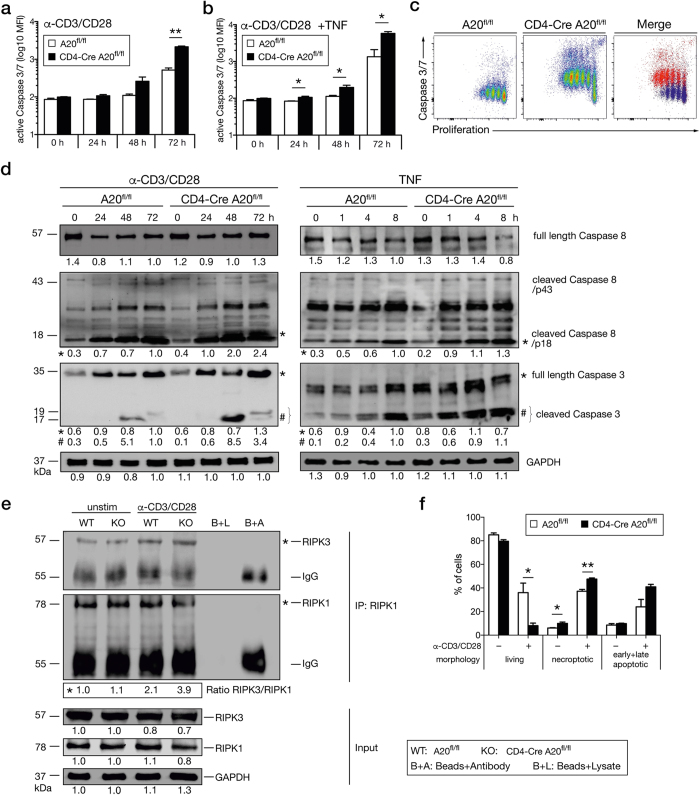
A20-deficient T cells are highly susceptible to apoptosis and necroptosis *in vitro.* CD8^+^ T cells from CD4-Cre A20^fl/fl^ and A20^fl/fl^ control mice were isolated and stimulated with anti-CD3/CD28 and/or TNF, respectively, for the indicated time points. (**a,b**) Active caspase-3/7 was determined by flow cytometry using DEVD substrate after stimulation with (**a**) anti-CD3/CD28 only, or (**b**) anti-CD3/CD28 with TNF. (**c**) Dot Plots showing proliferation and caspase-3/7 activity after 72 h of stimulation. (**d**) Western blot analysis of full length caspase-8, cleaved caspase-8, full-length caspase-3 and cleaved caspase-3 after TCR or TNF stimulation, respectively. (**e**) Lysates from unstimulated and 48 h anti-CD3/CD28-stimulated CD8^+^ T cells were immunoprecipitated with anti-RIPK1. Protein concentrations were equalized by staining lysates for GAPDH. Beads plus lysate without antibody (B+L) and beads plus anti-RIPK1 without lysates (B+A) were used as controls. (**f**) CD8^+^ T cells were left untreated or stimulated with anti-CD3/CD28 for 72 h and IFC was performed. A representative of 2 independent experiments is shown, with 3 mice per group. Error bars indicate + SEM. Student’s *t*-test, *p < 0.05; **p < 0.01.

Upon stimulation with anti-CD3/CD28, A20-competent and -deficient CD8^+^ T cells showed an increase in active caspase-3/7. This enhancement was significantly augmented in A20-deficient CD8^+^ T cells 72 h post stimulation (Fig. 5a). Upon additional stimulation with TNF, differences in active caspase-3/7 became significantly different even within 24 h (Fig. 5b). Notably, A20-deficient proliferating CD8^+^ T cells were characterized by increased caspase-3/7 activity compared to A20-competent CD8^+^ T cells (Fig. 5c).

To further confirm that A20-deficiency augmented apoptosis of CD8^+^ T cells upon *in vitro* stimulation, we analyzed procaspase-3 and −8 cleavage by western blotting in T cells activated by anti-CD3/CD28 or TNF, respectively. As illustrated in Fig. 5d, the amounts of cleaved effector caspase-3 as well as procaspase-8 cleavage products p43 and p18 were enhanced in A20-deficient CD8^+^ T cells upon anti-CD3/CD28 and TNF stimulation, respectively.

To study the necroptosis pathway, we analyzed RIPK1/RIPK3 interactions. Upon stimulation with anti-CD3/CD28, RIPK1 immunoprecipitates of A20-competent and –deficient CD8^+^ T cells showed presence of RIPK3 (Fig. 5e) illustrating the formation of the RIPK1/RIPK3 necroptotic complexes and that, in addition to apoptosis, necroptosis is induced in anti-CD3/CD28-activated CD8^+^ T cells.

To further analyze the regulation of cell death pathways by A20, we quantified the number of necroptotic and apoptotic CD8^+^ T cells by IFC analysis (Fig. 5f). In unstimulated cells the frequency of necroptotic, but not apoptotic, CD8^+^ T cells was increased in A20-deficient cells, corresponding to the RIPK1/RIPK3 complex formation and the absence of cleaved caspase-3 and −8 in A20-deficient CD8^+^ T cells (Fig. 5d,e). Upon anti-CD3/CD28 stimulation, necroptosis and apoptosis were strongly induced in A20-sufficient CD8^+^ T cells, but were even more prominent in A20-deficient CD8^+^ T cells, indicating that A20 regulates both cell death pathways.

### A20 inhibits CD95 expression and CD95L-induced apoptosis of CD8^+^ T cells

CD95 can contribute to the induction of two opposing processes: the activation or cell death of pathogen-specific CD8^+^ T cells. Among other factors the outcome of CD95 ligation depends on (i) the status of the T cell, i.e. naïve, resting or activated T cell, (ii) the amount of CD95 and CD95 agonists and (iii) the strength of CD95 signaling^37^,^39^,^40^,^41^,^42^,^43^. Therefore, we analyzed whether Lm OVA-specific CD8^+^ T cells upregulated CD95 during listeriosis and whether this process was regulated by A20.

**Figure 6:**
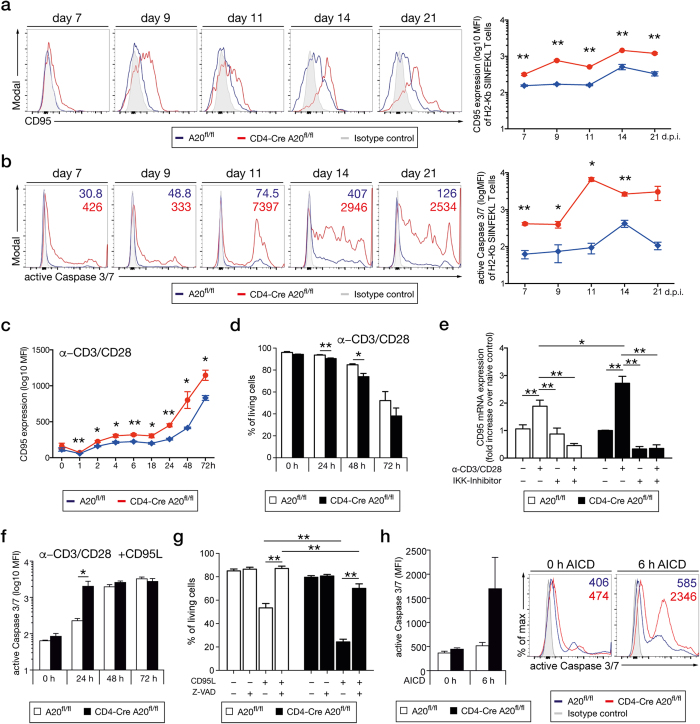
A20 reduces CD95 expression by inhibition of NF-κB activation. (**a**) Histograms display MFI of CD95 *ex vivo* on Lm OVA-specific CD8^+^ T cells at the indicated time points p.i. (**b**) *Ex vivo* kinetic of active caspase-3/7 in Lm OVA-specific CD8^+^ T cells at the indicated time points p.i. (**c**) CD8^+^ T cells were *in vitro* stimulated with anti-CD3/CD28 and flow cytometric analysis for CD95 was performed at the indicated time points. (**d**) Frequency of living cells was determined after *in vitro* stimulation with anti-CD3/CD28. Cell death was determined by flow cytometry, staining for annexin V and 7-AAD. (**e**) T cells were stimulated with anti-CD3/CD28, treated with IKK-inhibitor or were left untreated. mRNA was isolated and levels of CD95 were determined. (**f**) CD8^+^ T cells were stimulated *in vitro* with anti-CD3/CD28 and recombinant CD95L for the indicated time points. Active caspase 3/7 was measured using DEVD substrate. (**g**) Frequency of living cells was determined after CD8^+^ T cells were stimulated with CD95L for 2 h. To inhibit apoptosis, Z-VAD-FMK was added prior to the stimulation. (**h**) Active caspase-3/7 was determined before and after AICD. (**a,b**) Cells were gated on singlets and H2-Kb SIINFEKL^+^ CD8^+^ T cells. A representative of 2 independent experiments is shown, with 3 mice per group. Error bars indicate + SEM. (**a–d**,**f**,**h**) Student’s *t*-test. (**e,g**) Tukey’s multiple comparison test; *p < 0.05; **p < 0.01.

Already at day 7 p.i., A20-deficient Lm OVA-specific CD8^+^ T cells expressed more CD95 than A20-competent T cells. In both genotypes, CD95 expression increased over time but was significantly higher in A20-deficient pathogen-specific CD8^+^ T cells at all time points up to day 21 p.i. (Fig. 6a). In parallel to CD95 upregulation, caspase-3/7 activity was significantly upregulated in A20-deficient Lm OVA-specific CD8^+^ T cells at the peak of the primary CD8^+^ T cell response at day 7 p.i, increased drastically on day 11 p.i. and was maintained on high levels at all later time points. In contrast, only a slight increase of caspase-3/7 activity was observed in A20^fl/fl^ control mice (Fig. 6b).

Furthermore, *in vitro* stimulation of naïve CD8^+^ T cells with anti-CD3/CD28 induced a significantly stronger upregulation of CD95 in A20-deficient as compared to A20-sufficient CD8^+^ T cells (Fig. 6c). In parallel, numbers of living annexinV^−^/7-AAD^−^ CD8^+^ T cells declined in both groups but the reduction was significantly stronger in the absence of A20 (Fig. 6d).

Activation of NF-κB induces expression of CD95 and, thus, the increased cell death of A20-deficient CD8^+^ T cells may be partially mediated by augmented CD95 expression due to the lack of NF-κB inhibition^44^. To test this hypothesis, we analyzed the role of A20 for NF-κB-mediated induction of CD95 expression in CD8^+^ T cells. Upon stimulation with anti-CD3/CD28 A20-deficient CD8^+^ T cells CD95 mRNA increased 2.7 fold, whereas in A20-sufficient CD8^+^ T cells upregulation of CD95 mRNA was only 1.8 fold (Fig. 6e). Importantly, inhibition of NF-κB activation by an IKK-inhibitor prevented upregulation of CD95 mRNA in both A20-deficient and -sufficient cells and completely abolished differences between the two genotypes (Fig. 6e).

To test the functional importance of the increased NF-κB-dependent CD95 expression of A20-deficient CD8^+^ T cells, we treated anti-CD3/CD28-stimulated CD8^+^ T cells with CD95L. In fact, CD95L stimulation induced a significantly stronger activation of caspase-3/7 in A20-deficient CD8^+^ T cells within 24 h (Fig. 6f). Noteworthy, inhibition of the caspase activity by Z-VAD-FMK significantly reduced cell death of A20-deficient and competent CD95L-stimulated CD8^+^ T cells (Fig. 6g) illustrating that CD95-induced apoptosis of activated CD8^+^ T cells is strongly reduced by A20. However, even in Z-VAD-FMK-treated cells, the frequency of living cells was still significantly reduced in A20-deficient cells, indicating that in addition to apoptosis, other cell death pathways, e.g. necroptosis, are augmented in CD95-stimulated A20-deficient CD8^+^ T cells.

*In vivo*, CD95-mediated activation induced cell death (AICD) plays a critical role in the contraction phase of pathogen-specific T cells to maintain immune homeostasis after clearance of the infection^37^. To study whether A20 regulates AICD, we stimulated CD8^+^ T cells with anti-CD3/CD28 and IL-2 for 2 days, expanded the cells in IL-2 containing medium for another 3 days before restimulation with anti-CD3. As illustrated in Fig. 6h, anti-CD3 restimulation induced a much stronger caspase-3/7 activity in A20-deficient CD8^+^ T cells compared to A20-competent cells demonstrating that A20 suppresses AICD.

Taken together, these data delineate a crucial inhibitory role of A20 on CD95-induced CD8^+^ T cell apoptosis.

## Discussion

The control of intracellular infections requires complex dynamics of protective pathogen-specific CD8^+^ T cells including (i) the expansion of effector CD8^+^ T cells upon primary infection, (ii) the contraction of these effector T cells after pathogen clearance, (iii) the development and persistence of memory CD8^+^ T cells, and (iv) the rapid expansion of memory CD8^+^ upon reinfection. Here, we show that A20 regulates all of these different CD8^+^ T cell responses and plays an opposing role for pathogen-specific CD8^+^ T cell responses in the course of an intracellular bacterial infection. At the peak of the primary CD8^+^ T cell response (day 7 p.i.), the A20-deficient effector CD8^+^ T cell response was greatly augmented with increased numbers of IFN-γ-producing and cytotoxic granzyme B^+^ CD8^+^ T cells resulting in an improved clearance of the pathogen. Thereafter, A20-deficient pathogen-specific CD8^+^ T cells failed to establish a protective memory T cell pool, resulting in a diminished control of *Listeria* upon secondary infection.

Recently, Drennan *et al*. demonstrated that splenic CD8^+^ T cells of naïve mice express A20 *in vivo*^32^. In accordance with Giordano *et al*.^31^, we also observed a low baseline expression of A20 in unstimulated CD8^+^ T cells *in vitro*, which was upregulated in TCR-stimulated CD8^+^ T cells, and restricted activation of NF-κB and MAP kinases, CD8^+^ T cell proliferation, and IFN-γ and TNF production. Of note, numbers of IFN-γ-producing as well as granzyme B^+^ pathogen-specific CD8^+^ T cells were significantly increased in CD4-Cre A20^fl/fl^ mice at the peak of the primary T cell response. In naïve CD4-Cre A20^fl/fl^ mice, the absence of this suppressive activity of A20 may account for the increased activation of CD4^+^ and CD8^+^ T cells as evidenced by elevated CD69 expression. Of note, the increased activation of T cells, which has also been detected by Giordano *et al*.^31^, did not result in a spontaneous inflammatory disease of CD4-Cre A20^fl/fl^ mice (our unpublished data), which has also not been described before^31^,^32^. This may be explained by the fact that cytokine production of CD4^+^ (IFN-γ, IL-2) and CD8^+^ T cells (IFN-γ, granzyme B) was not increased in naïve CD4-Cre as compared to A20^fl/fl^ mice. This is in contrast to mouse strains with deletion of A20 in B cells, dendritic cells and macrophages, respectively, which all develop severe spontaneous inflammatory diseases^23^,^24^,^25^,^26^,^45^ and indirectly argues for a specific deletion of A20 in T cells of CD4-Cre A20^fl/fl^ mice.

Although activation of NF-κB is required for the development of an efficient primary effector CD8^+^ T cell response as well as for the development of memory T cells^4^,^7^,^46^, the excessive and prolonged activation of this signaling pathway can either lead to the development of autoimmunity or the induction of programmed cell death^47^. We considered two major hypotheses, how the increased NF-κB activation of A20-deficient pathogen-specific CD8^+^ T cells might cause reduced numbers of T_EM_ and T_CM_: (i) A20 regulates the development of SLEC and MPEC CD8^+^ T cells, and (ii) A20 inhibits cell death and enables persistence of CD8^+^ T cells. A detailed analysis of the *in vivo* kinetics of Lm-specific T cells revealed that absolute numbers of Lm-specific CD8^+^ T_CM_ and T_EM_, respectively, were increased in CD4-Cre A20^fl/fl^ mice at the peak of the primary CD8^+^ T cell response. However already at day 9 p.i., absolute numbers of Lm OVA-specific CD8^+^ T cells were reduced in CD4-Cre A20^fl/fl^ mice due to an increased cell death of A20-deficient T cells. In particular, the maintenance of MPEC was severely impaired in A20-deficient CD8^+^ T cells, which might contribute to the impaired pathogen-specific CD8^+^ T_EM_ and T_CM_ response of CD4-Cre A20^fl/fl^ mice. Noteworthy, Lm OVA-specific CD8^+^ T cells increased 15-fold upon reinfection (Fig. 3h,i), whereas A20-deficient Lm OVA-specific CD8^+^ T cells increased only 3-fold within three days after reinfection. Although this marginal increase may indicate an impaired expansion of A20-deficient Lm-specific CD8^+^ T cells, this assumption has to be regarded with caution, since the significantly lower number of pathogen-specific A20-deficient memory CD8^+^ T cells at day 50 p.i. will result *per se* and A20-independent in lower numbers of pathogen-specific CD8^+^ T cell three days after reinfection. In addition to the development and expansion of CD8^+^ T cell subsets, A20 regulated the functional activity of Lm OVA-specific CD8^+^ T cells. At the peak of the primary CD8^+^ T cell response, IFN-γ and granzyme B production of A20-deficient CD8^+^ T cells was augmented, whereas it was diminished thereafter and also upon reinfection. This corresponds to the increased absolute number of living A20-deficient Lm OVA-specific CD8^+^ T cells at day 7 p.i. and the reduced number of A20-deficient pathogen-specific CD8^+^ T cells from day 9 p.i onward (Fig. 3d). At day 7 p.i., the co-inhibitory receptor PD-1 was equally induced in both A20-deficient competent and –deficient Lm OVA-specific CD8^+^ T cells, which is consistent with the induction of this inhibitory receptor during CD8^+^ T cell activation^48^. However, in contrast to A20-competent CD8^+^ T cells, A20-deficient Lm OVA-specific CD8^+^ T cells failed to downregulate and even further upregulated PD-1 after the elimination of *Listeria*. We speculate that this elevated PD-1 expression of A20-deficient CD8^+^ T cells contributed to their functional impairment, since PD-1 causes the characteristic exhausted phenotype, i.e. poor cell division and cytokine production of CD8^+^ T cells in chronic viral infections^49^. At present we do not know whether A20 reduces PD-1 expression by a direct or indirect mechanism but so far it has not been reported that A20 regulates the transcriptional repressor Blimp-1, which directly silences PD-1 expression in the late phases of acute effector CD8^+^ T cell responses^50^.

CD8^+^ T cell memory and SLEC development and, thereby, protective secondary CD8^+^ T cell responses are dependent on CD4^+^ T cell help in listeriosis^8^,^9^,^34^. Since the frequency of Lm-specific IFN-γ^+^ and IL-2^+^ CD4^+^ T cells were not affected by A20-deficiency in primary and secondary listeriosis, and, additionally, CD25 expression of CD8^+^ T cells was upregulated normally in CD8^+^ T cells, a diminished CD4^+^ T cell response is unlikely to cause the A20-dependent effects in CD8^+^ T cells. Although we do not formally show that the defective CD8^+^ T cell response of A20-deficient CD8^+^ T cells is only caused by the intrinsic A20-defect, these *in vivo* data in combination with the corresponding *in vitro* phenotype of A20-deficient CD8^+^ T cells strongly suggest that A20 expression in CD8^+^ T cells is the major factor causing the altered CD8^+^ T cell response in listeriosis.

Apoptosis and necroptosis are two different forms of programmed cell death, which can be induced in T cells by stimulation with TNF^51^ and activation of the death receptor CD95^52^. Whereas apoptosis is mediated by the activation of caspases including caspase-3 and −7, necroptosis depends on the formation and activation of a RIPK1/RIPK3 complex^53^. Several studies suggest that the contraction of pathogen-specific CD8^+^ T cells after primary infection is caused by apoptosis, which is in part dependent on CD95 stimulation^54^,^55^. However, quantitative *in vivo* data on the respective role of apoptosis and necroptosis for the contraction of pathogen-specific CD8^+^ T cells have not been reported so far, which is largely based on the difficulty to unequivocally differentiate and quantify apoptosis and necroptosis *in vivo*. Of note, apoptosis and necroptosis are morphologically distinct. Apoptotic cells are characterized by phosphatidylserine exposure on the cell surface followed by membrane blebbing, nuclear fragmentation, decreased cellular volume and formation of apoptotic bodies^56^. In contrast, necroptotic cells include early plasma membrane rupture followed by rapid cytoplasmic and nuclear swelling and organelle breakdown^38^. Based on these differences, we used IFC, which combines quantitative single cell microscopy with flow cytometry and quantified *ex vivo* apoptotic and necroptotic pathogen-specific CD8^+^ T cells. Of note, this analysis included all OVA_257–264_-specific CD8^+^ T cells including living, apoptotic and necroptotic cells.

In A20-competent pathogen-specific CD8^+^ T cells, necroptosis and apoptosis equally contributed to the cell death and contraction of the Lm OVA-specific CD8^+^ T cell pool. In A20-deficient pathogen-specific CD8^+^ T cells necroptosis was enhanced but apoptosis was decreased at the peak of the T cell response (day 7 p.i.), indicating a role of A20 as a suppressor of necroptosis. However, at day 11 p.i. more apoptosis and necroptosis of Lm-specific CD8^+^ T cells were observed in CD4-Cre A20^fl/fl^ mice. Thus, A20 inhibits both cell death pathways in pathogen-specific CD8^+^ T cells *in vivo*, although the relative effect of A20 on each of these two pathways may be regulated by additional factors including CD95 expression (Fig. 6a).

The FlowSight analysis of Lm OVA-specific CD8^+^ T cells (Fig. 4) revealed that (i) the total number of H2-Kb SIINFEKL-specific CD8^+^ T cells as displayed in Figs 2a and 3b was composed of living, apoptotic and necroptotic cells and (ii) the relative numbers of necroptotic and/or apoptotic pathogen-specific CD8^+^ T cells were increased and relative numbers of living cells reduced in CD4- Cre A20^fl/fl^ mice (Fig. 4f). The increased numbers of apoptotic/necroptotic A20-deficient pathogen-specific CD8^+^ T cells explain why the initially significantly elevated absolute numbers of A20-deficient Lm OVA-specific CD8^+^ T cells (day 7 p.i.) rapidly declined and were already significantly reduced at day 11 p.i. as compared to A20-competent CD8^+^ T cells. Only at day 7 p.i., the absolute numbers of living A20-deficient Lm OVA-specific CD8^+^ T cells were increased as compared to A20-competent CD8^+^ T cells, which coincides with the increased absolute numbers of IFN-γ and granzyme B producing pathogen-specific CD8^+^ T cells and the significantly reduced bacterial load in CD4-Cre A20^fl/fl^ mice at this time point.

A recent *in vitro* study of Onizawa *et al*. also identified A20 as a suppressor of necroptosis in CD4^+^ T cells acting at ubiquitination of RIPK3^21^. However, in contrast to our data on pathogen-specific CD8^+^ T cells, A20-deficiency of CD4^+^ T cells completely abolished apoptosis. In contrast, Drennan *et al*. demonstrated that A20-deficiency resulted in an increased cell death of invariant NK T cells due to apoptosis but not necroptosis^32^. At present, the discrepant results concerning the induction and A20-mediated regulation of apoptosis and necroptosis of CD8^+^ (our study), CD4^+^ T cells^21^ and invariant NK T cells^32^ are unresolved but may be explained by intrinsic cell-type specific differences between these two T cell subsets.

Under *in vitro* conditions, flow cytometry and IFC in combination with western blot analysis of active caspase-3 and −8 and RIPK1/RIPK3 complex formation further illustrated that irrespective of A20 expression, TCR-stimulated CD8^+^ T cells underwent both apoptosis and necroptosis. A20-deficient CD8^+^ T cells were more prone to necroptosis, which is mediated via RIPK1/RIPK3 complex formation. A20 removes Lys63 chains from RIPK1 *via* its OTU domain and promotes the addition of degradative polyubiquitin chains on RIPK1 *via* its zinc-finger 4 motif. Deubiquitination of RIPK1/RIPK3 complex is a key step towards necroptosis induction, therefore, the molecular mechanisms of the effects observed in our work require further intensive investigations. Furthermore, upon *in vitro* stimulation with anti-CD3/CD28, both necroptosis and apoptosis were increased in A20-deficient CD8^+^ T cells.

T cell apoptosis can be induced by several factors including TNF and CD95L. In accordance, stimulation with TNF induced active caspase-3 and −8 and stimulation with CD95L mediated active caspase-3/7 expression in naïve CD8^+^ T cells *in vitro*. Importantly, both pathways were augmented in A20-deficient cells indicating that *in vivo* cell death of Lm-specific CD8^+^ T cells is most probably mediated by several mechanisms including TNF and CD95.

It has been shown before that the contraction and survival of pathogen-specific CD8^+^ T cells is independent of CD95 expression^57^,^58^, while the maintenance of functional Lm-specific CD8^+^ T cells requires CD95/CD95L interactions in a CD8^+^ T cell extrinsic manner^12^. However, an increased expression of CD95, as observed in mice with constitutive NF-κB activation^5^, may contribute to an increased cell death of Lm-specific CD8^+^ T cells, which is in line with CD95 as an apoptosis inducing factor upon NF-κB-mediated activation^44^. Corresponding to the increased NF-κB activation (Supplementary Fig. S4A), A20-deficient CD8^+^ T cells strongly upregulated CD95 upon activation and inhibition of NF-κB completely abolished the increased CD95 expression of TCR-stimulated A20-deficient CD8^+^ T cells. Collectively, the published and our data are compatible with the assumption that depending on the strength of CD95 stimulation and signaling, cell death of activated T cells may be induced^59^. In listeriosis, A20-deficient Lm OVA-specific CD8^+^ T cells expressed more CD95 and active caspase-3/7 as compared to A20-sufficient CD8^+^ T cells. Thus, the increased cell death of A20-deficient pathogen-specific CD8^+^ T cells may partially depend on CD95 in combination with other cell death inducing mechanisms including the TNF receptor pathway.

*In vivo*, pathogen-specific CD8^+^ T cells may encounter repetitively cognate-antigen presenting cells. Therefore, a balance between TCR-induced expansion and CD95-mediated AICD of CD8^+^ T cells is mandatory. Experimentally, AICD can be mimicked *in vitro* by repetitive TCR stimulation and, here, we demonstrate a significantly increased caspases-3/7 activity in A20-deficient CD8^+^ T cells upon AICD-induction. Thus, AICD will further contribute to the impaired survival of A20-deficient Lm-specific CD8^+^ T cells.

Taken together, A20 limits the magnitude of the primary pathogen-specific CD8^+^ T cell response but strengthens memory T cell survival and secondary CD8^+^ T cell responses by restricting the apoptosis- and necroptosis-mediated contraction of the pathogen-specific CD8^+^ T cell pool.

## Materials and Methods

### Mice

A20^fl/fl^ mice on a C57BL/6 background as described before were crossed with CD4-Cre mice to obtain CD4-Cre A20^fl/fl^ mice^25^. Genotyping was done by PCR of tail DNA with primers for CD4-Cre and A20^fl/fl^.

### Ethics statement

Animal experiments were performed according to the German Animal Welfare Act (Deutsches Tierschutzgesetz) and approved by local authorities (Landesverwaltungsamt Sachsen-Anhalt; file number: 42502-2-994). All efforts were made to minimize suffering and surgery was performed after euthanization.

### Infection of mice and determination of colony forming units

*Listeria monocytogenes* wild type (Lm WT, EGD strain) and the ovalbumin expressing strain *L. monocytogenes* (Lm OVA) were used in the experiments, respectively. *Listeria* were grown in brain heart infusion broth and aliquots of log-pase cultures were stored at −80 °C. Fresh log-phase cultures were prepared from frozen stocks and mice were infected i.v. *via* the tail vein with 1 × 10^4^ Lm WT and 5 × 10^4^ Lm OVA, respectively, diluted in 200 μl sterile PBS (pH 7.4). Reinfection was performed with 1 × 10^6^ CFU Lm WT or Lm OVA, respectively, at day 50 p.i. Bacterial dose used for infections was confirmed by plating an inoculum on brain heart infusion agar and counting colonies after incubation at 37 °C for 24 h.

To determine CFUs in infected mice, animals were sacrificed at the indicated time points p.i., spleens were homogenized with sterile tissue grinders. Tenfold serial dilutions of the homogenates were plated on brain heart infusion agar. Bacterial colonies were counted after incubation at 37 °C for 24 h and the number of bacteria per spleen was calculated.

### Flow cytometry

Single cell suspensions from spleen, liver, lymph nodes and thymus were prepared as described before^11^. Cells were stained for 30 min at 4 °C using mouse specific fluorochrome-labled antibodies for CD3 (145-2C11), CD4 (RM4-5), CD8 (53-6.7), CD45 (30-F11) for T cells, F4/80 (BM8), CD11b (M1/70), Ly-6G (1A8) and Ly-6C (HK1.4) for macrophages, neutrophils and monocytes, CD11c (N418) for dendritic cells and NK1.1 (PK136) for NK cells from BioLegend or eBioscience after blocking with anti-CD16/32 (BioLegend). Ag-specific CD8^+^ T cells were detected using PE-conjugated H2-Kb SIINFEKL pentamer (ProImmune). CD8^+^ T cells were further characterized by staining with antibodies for CD69, CD62L (MEL-14), CD44 (IM7), CD127 (A7R34), KLRG-1 (2F1/KLRG1), PD-1 (RMP1-14) and CD95 (SA367H8). Intracellular staining was performed by *ex vivo* stimulation for 4 h at 37 °C with the ovalbumin-specific peptide H-SIINFEKL-OH (SIINFEKL) for CD8^+^ T cells or H-ISQAVHAAHAEINEAGR-OH (OVACD4) for CD4^+^ T cells in presence of Brefeldin A (BioLegend) and stained for IFN-γ (XMG1.2), granzyme B (NGZB) or IL-2 from BioLegend with the Intracellular Fixation & Permeabilization Kit from eBioscience. Cytokine concentration in supernatant and serum was determined using cytometric bead array (BD Bioscience). Active caspase-3/7 measurement was performed using CellEvent Caspase-3/7 Green ReadyProbes (Life Technologies). Flow cytometric analysis was performed on a BD FACS CANTO II (BD Bioscience) and analyzed with FlowJo X software (Milltenyi).

### *In vitro* T cell stimulation

CD8^+^ T cells were isolated from spleen and lymph nodes from A20^fl/fl^ and CD4-Cre A20^fl/fl^ mice with Mouse CD8^+^ T cell Isolation Kit (Stemcell Technologies) according to manufacturer’s instruction. Cells were stimulated in RPMI 1640 medium supplemented with L-glutamine, 10% FCS, 100 U/ml penicillin, 100 μg/ml streptomycin, 1 mM sodium pyruvate, 1% non-essential amino acids, 50 μM β-mercaptoethanol (all from Life Technologies) and 5 mM HEPES (Biochrom) in the presence of 1 μg/ml plate-bound anti-CD3 and 2 μg/ml soluble anti-CD28 (both from BioLegend) for 3 days.

Proliferation was analyzed by staining cells with carboxyfluorescein succinimidyl ester (CFSE, BioLegend) before stimulation.

For active caspase-3/7 measurement, CD8^+^ T cells were stimulated for the indicated time points with the addition of 20 ng/ml TNF or recombinant CD95L (50 ng/ml).

### Activation induced cell death (AICD)

CD8^+^ T cells were isolated and stimulated in the presence of 1 μg/ml plate-bound anti-CD3, 2 μg/ml soluble anti-CD28 and 20 ng/ml IL-2. On day 2, cells were harvested and expanded in IL-2-containing medium for 3 days. On day 5, T cells were restimulated for 6 h with 10 μg/ml plate-bound anti-CD3.

### Imaging Flow Cytometry (IFC)

Spleen cells were isolated as described before and stained for H2-Kb SIINFEKL-specific CD8^+^ T cells. Cells were washed and resuspended in annexin staining buffer and stained with annexin V (Life Technologies) and 7-AAD (BioLegend).

For *in vitro* analysis cells were stimulated for three days with anti-CD3/CD28 or 2 h with CD95L, respectively. Staining was done as described above. IFC was performed on Amnis FlowSight Imaging Flow Cytometer (EMD Millipore).

### Western Blot analysis

Cells were isolated from spleen and lymph nodes, sorted for total T cells (Stemcell Technologies) and resuspended in radioimmunoprecipitation assay (RIPA) buffer with protease inhibitor after stimulation with anti-CD3/CD28 or TNF for the indicated time points. After 30 min incubation on ice the lysates were centrifuged and the supernatant was collected. Protein concentration was measured with Bradford Assay and was calculated based on a standard dilution using photometer. For SDS-PAGE, sample buffer was added to lysates in a 1:5 dilution and incubated for 5 min at 95 °C. After gel run proteins were transferred on PVDF membranes and afterwards incubated in TBST with 5% BSA or Blotto B. Antibodies against A20 (A-12) from Santa Cruz, p-IκBα (Ser32), p-p38 (Thr180/Tyr182), p-ERK (Thr202/Tyr204), Caspase-3 and Caspase-8 (cell signaling) were used. Blots were developed using ECL Plus Kit (Thermo Scientific).

### Immunoprecipitation

Proteins from unstimulated and anti-CD3/CD28-stimulated CD8^+^ T cells were isolated as described before and pre-incubated with Sepharose beads (GE Healthcare Bio-Sciences) for 30 min at 4 °C with agitation to block unspecific binding. Afterwards samples were centrifuged for 10 min at 10,000 × g. Equal amounts of protein were incubated with anti-RIPK1 antibody (cell signaling) over night at 4 °C on a rocker. Sepharose beads were added to the samples and incubated over night at 4 °C on a rocker. To collect the immunoprecipitate, the samples were centrifuged and the beads were washed 3 times with PBS to remove unspecific binding. After the last washing step, beads were resuspended in 1X lane marker reducing sample buffer and incubated at 99 °C for 3 min. The beads were removed by centrifugation and western blot analysis was performed as described above.

### RT-qPCR

T cells were stimulated for the indicated time points and total RNA was isolated according to manufacturer’s instruction (RNeasy kit, Qiagen). cDNA synthesis was generated using SuperScript reverse transcriptase kit (Invitrogen) and qPCR was performed for HPRT and CD95 with TaqMan probes (Applied Biosystems) on a Lightcycler 480 system (Roche). Data were analyzed according to ΔΔ cycle threshold method.

### Statistical analysis

All data are represented as mean + SEM and statistical analysis was performed as indicated in the figure legends, respectively, using GraphPad Prism 5 software. A p value of <0.05 was considered statistically significant.

## Additional Information

**How to cite this article**: Just, S. *et al*. A20 Curtails Primary but Augments Secondary CD8^+^ T Cell Responses in Intracellular Bacterial Infection. *Sci. Rep.* **6**, 39796; doi: 10.1038/srep39796 (2016).

**Publisher's note:** Springer Nature remains neutral with regard to jurisdictional claims in published maps and institutional affiliations.

## Supplementary Material

Supplementary Material
