# Supplementary material for: A20 Curtails Primary but Augments Secondary CD8+ T Cell Responses in Intracellular Bacterial Infection
Source: Sci Rep. 2016 Dec 22;6:39796. doi: 10.1038/srep39796 (PMC5177869; doi:10.1038/srep39796)
Supplement: Supplementary Material [file srep39796-s1.doc]

**A20 Curtails Primary but Augments Secondary CD8^+^ T Cell Responses in Intracellular Bacterial Infection**

Sissy Just^1^, Gopala Nishanth^1,2^, Jörn H. Buchbinder^3^, Xu Wang^1^, Michael Naumann^4^, Inna Lavrik^3^, Dirk Schlüter*^1,2^

^1^Institute of Medical Microbiology and Hospital Hygiene, Otto-von-Guericke University Magdeburg, 39120 Magdeburg, Germany;

^2^Organ-specific Immune Regulation, Helmholtz-Center for Infection Research, 38124 Braunschweig, Germany;

^3^Department of Translational Inflammation Research, Otto-von-Guericke University Magdeburg, 39106 Magdeburg, Germany;

^4^Institute of Experimental Internal Medicine, Otto-von-Guericke University Magdeburg, 39120 Magdeburg, Germany

*Correspondence:

Prof. Dr. Dirk Schlüter

Tel.: 0391-67-13393

Fax: 0391-67-13384

Email: [dirk.schlueter@med.ovgu.de](mailto:dirk.schlueter@med.ovgu.de)


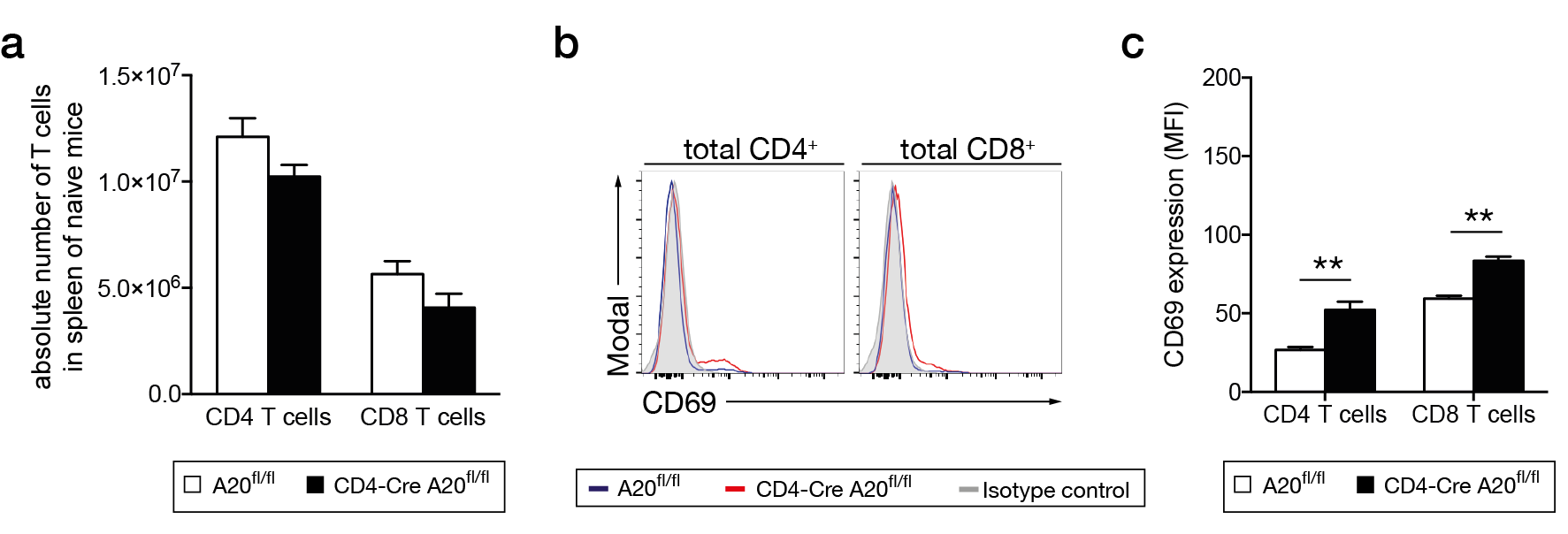


**Supplementary Figure S1:**

(A) Splenocytes from 8-week-old naïve mice were isolated and absolute number of CD4^+^ and CD8^+^ T cells was determined. (B) Representative histograms of CD69 expression on CD4^+^ and CD8^+^ T cells in spleens of naïve mice. (C) MFI of CD69 on T cells was determined. Error bars indicate +SEM; Student's *t*-test * p< 0.05; ** p< 0.01.


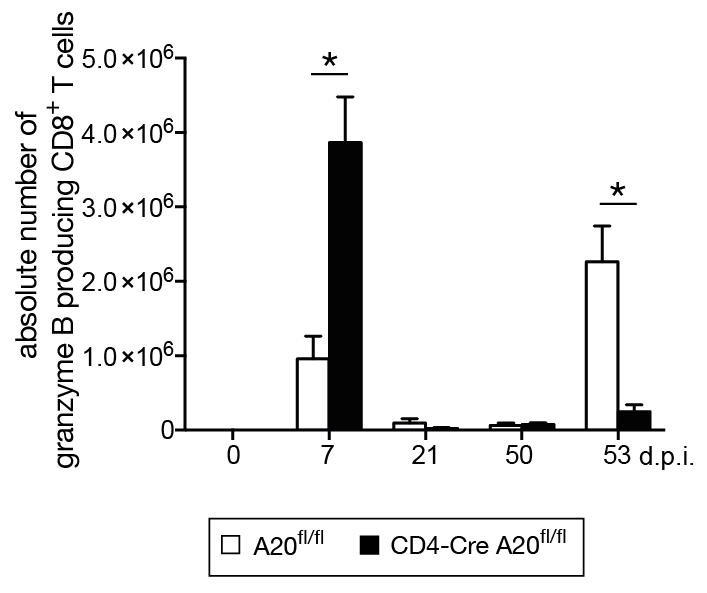


**Supplementary Figure S2: Absolute numbers of granzyme B-producing CD8^+^ T cells.**

CD4-Cre A20^fl/fl^ and A20^fl/fl^ control mice were infected with a non-lethal dose of Lm OVA and absolute numbers of granzyme B producing CD8^+^ T cells were determined at the indicated timepoints p.i. A representative of 3 independent experiments is shown with 3 mice per group. Error bars indicate +SEM. Student's *t*-test, * p< 0.05.


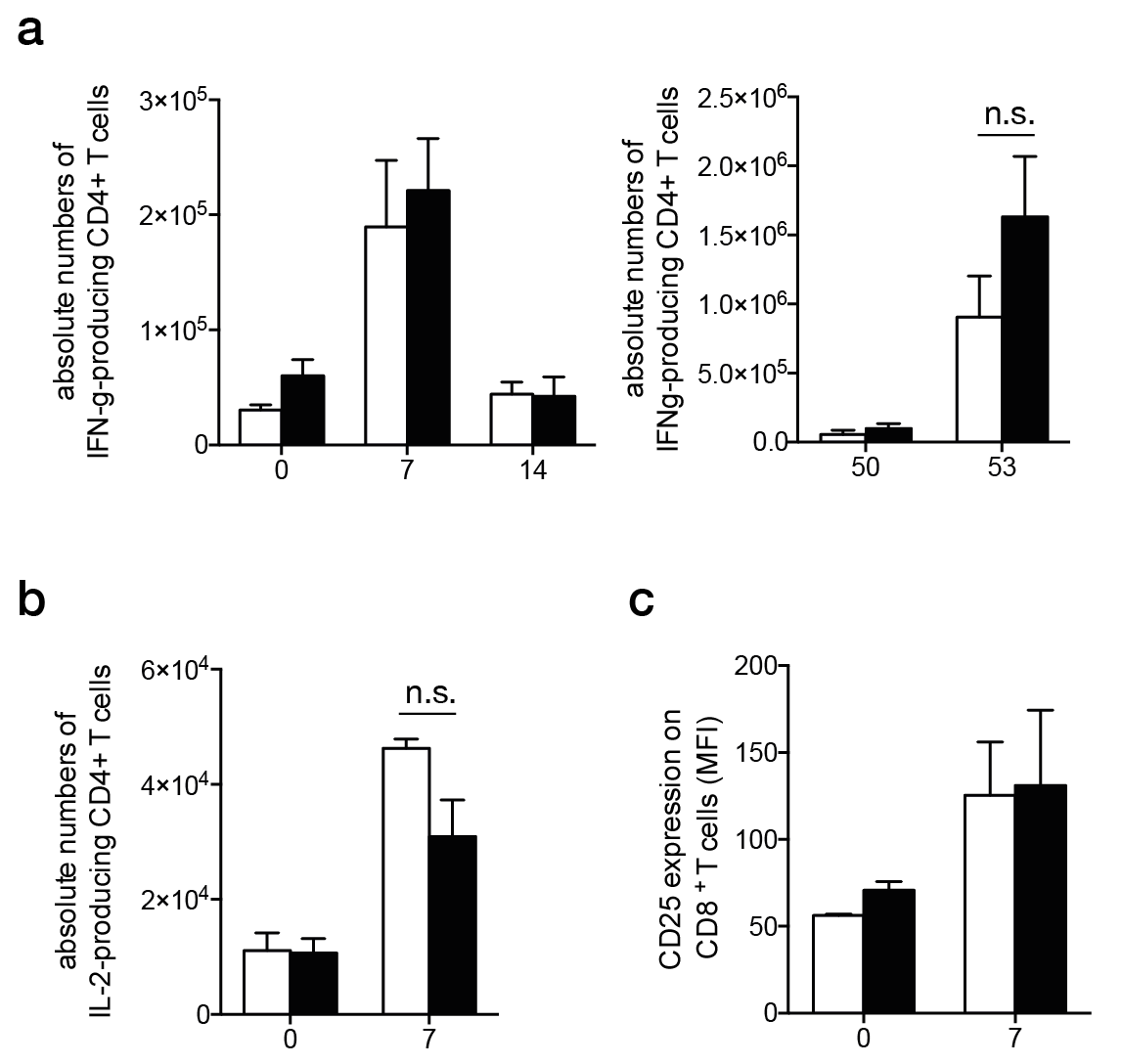


**Supplementary Figure S3: Primary and secondary CD4^+^ T cell response is not impaired in CD4-Cre A20^fl/fl^ mice.**

CD4-Cre A20^fl/fl^ and A20^fl/fl^ control mice were infected with a non-lethal dose of Lm OVA and CD4^+^ T cell response in spleen was analyzed at the indicated timepoints. (A) Absolute number of IFN-γ-producing CD4^+^ T cells at day 0, 7 and 14 p.i. after restimulation with OVACD4. (B) Absolute number of IFN-γ producing CD4^+^ T cells at day 50 and 3 days after reinfection, at day 53. (C) Absolute number of IL-2-producing CD4^+^ T cells at day 0 and 7 p.i. (D) Expression of CD25 on CD8^+^ T cells at day 0 and 7 p.i. A representative of 2 independent experiments is shown with 3 mice per group. Error bars indicate +SEM. Student's *t*-test, n.s. not significant.


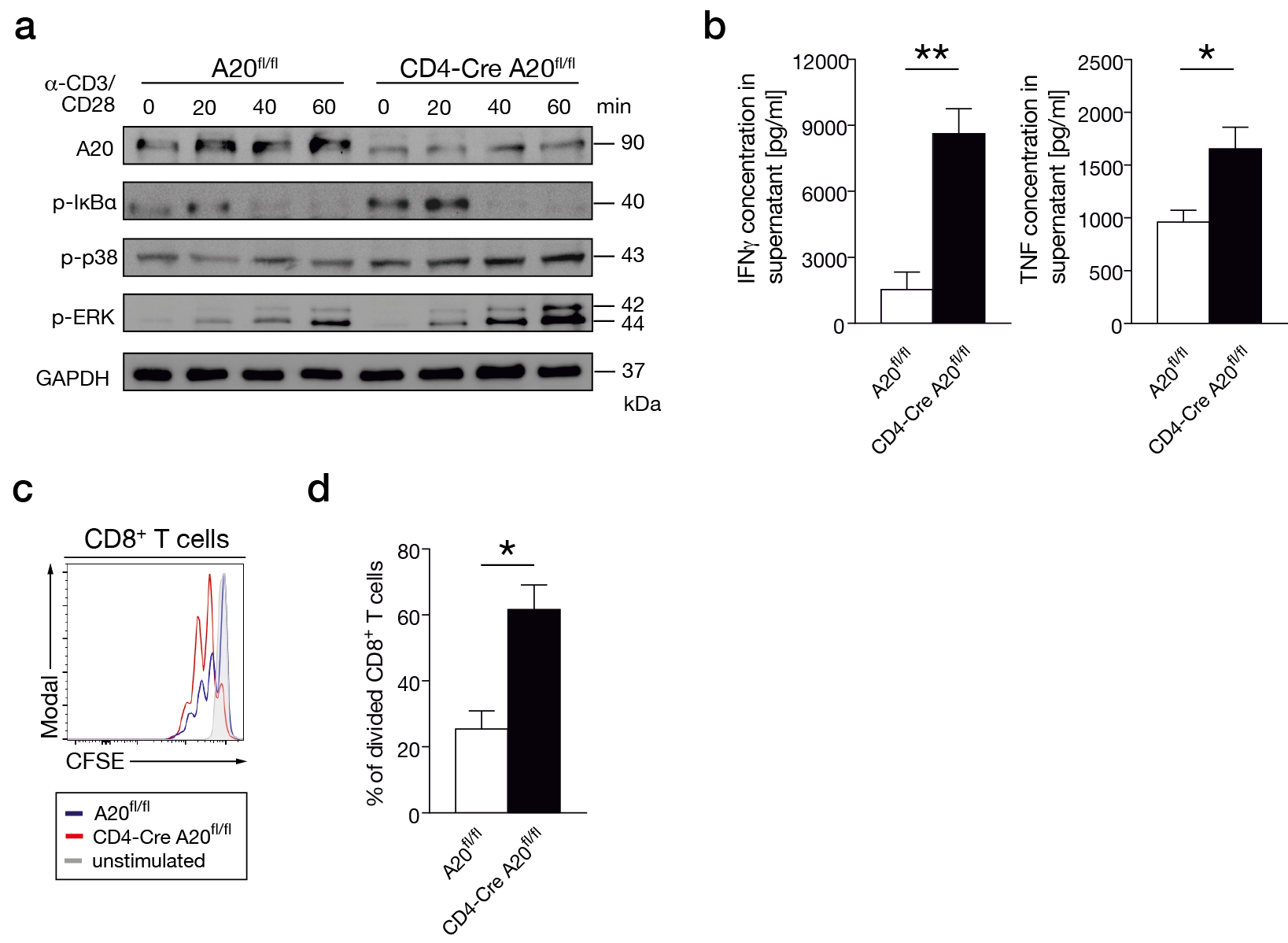


**Supplementary Figure S4: Increased activation and proliferation of A20-deficient CD8^+^ T cells *in vitro*.**

CD8^+^ T cells from naïve mice were isolated and cultivated in the presence of 1 µg/ml plate-bound anti-CD3 and 2 µg/ml soluble anti-CD28. (A) Proteins from T cells were isolated after stimulation for 0, 20, 40 or 60 min. WB was performed using antibodies against A20, p-IκBα, p-p38, p-ERK and GAPDH. (B) CD8^+^ T cells were stimulated for 72 h and supernatant was obtained to measure the concentration of IFN-γ and TNF by CBA. (C) To measure the proliferative activity of CD8^+^ T cells, cells were labeled with CFSE and stimulated for 72 h. (D) Frequency of divided cells after stimulation. A representative of 2 independent experiments is shown, with 3 mice per group. Error bars indicate +SEM. Student's *t*-test, * p< 0.05; ** p< 0.01.
